# Neonatal Imitation: Theory, Experimental Design, and Significance for the Field of Social Cognition

**DOI:** 10.3389/fpsyg.2017.01323

**Published:** 2017-08-04

**Authors:** Stefano Vincini, Yuna Jhang, Eugene H. Buder, Shaun Gallagher

**Affiliations:** ^1^Instituto de Investigaciones Filosóficas, Universidad Nacional Autónoma de México Mexico City, Mexico; ^2^Department of Speech Language Pathology and Audiology, Chung Shan Medical University Taichung, Taiwan; ^3^School of Communication Sciences and Disorders, University of Memphis, Memphis TN, United States; ^4^Institute for Intelligent Systems, University of Memphis, Memphis TN, United States; ^5^Department of Philosophy, University of Memphis, Memphis TN, United States; ^6^Faculty of Law, Humanities and the Arts, University of Wollongong, Wollongong NSW, Australia

**Keywords:** neonate imitation, arousal, active intermodal matching (AIM), association by similarity (AST), experimental methodology, social cognition

## Abstract

Neonatal imitation has rich implications for neuroscience, developmental psychology, and social cognition, but there is little consensus about this phenomenon. The primary empirical question, whether or not neonatal imitation exists, is not settled. Is it possible to give a balanced evaluation of the theories and methodologies at stake so as to facilitate real progress with respect to the primary empirical question? In this paper, we address this question. We present the operational definition of differential imitation and discuss why it is important to keep it in mind. The operational definition indicates that neonatal imitation may not look like prototypical imitation and sets non-obvious requirements on what can count as evidence for imitation. We also examine the principal explanations for the extant findings and argue that two theories, the arousal hypothesis and the Association by Similarity Theory, which interprets neonatal imitation as differential induction of spontaneous behavior through similarity, offer better explanations than the others. With respect to methodology, we investigate what experimental design can best provide evidence for imitation, focusing on how differential induction may be maximized and detected. Finally, we discuss the significance of neonatal imitation for the field of social cognition. Specifically, we propose links with theories of social interaction and direct social perception. Overall, our goals are to help clarify the complex theoretical issues at stake and suggest fruitful guidelines for empirical research.

## Introduction

There is a presumed phenomenon, well circumscribed but particularly intriguing, over which the science of embodied intersubjectivity has to take a position. This phenomenon concerns the first 2 months of post-natal life and is labeled “neonatal imitation” (NI), giving the term “neonatal” an unusually wide sense (Meltzoff and Moore, 1997; Nagy et al., 2013; Simpson et al., 2014; Oostenbroek et al., 2016).^1^ The questions of whether and in what sense infants in that early stage imitate facial gestures have considerable implications. For example, if NI actually occurs, resistance toward the idea that the newborn’s brain may regulate responses that match different visual models (Keven and Akins, 2016) will have to be revised. If NI is real, the hypothesis that imitation develops through associations via contiguity and contingency (Ray and Heyes, 2011) is seriously undermined if not altogether falsified. And if imitation is real but the findings do not justify postulating a foundational socio-cognitive role for the “recognition” of self-other similarities, then Meltzoff and Moore’s (1997) influential theory should be called into question on this point. Thus, although it is unlikely that a widespread agreement will be reached soon, it is worth striving toward a solution to the questions surrounding NI as they have significant repercussions for neuroscience, developmental psychology, and social cognition.

In this paper, our goal is to contribute to both theory and empirical methodology. In Section “The Operational Definition and the Importance of Keeping it in Mind,” we present the operational definition of NI as differential imitation and discuss why it is important to keep it in mind. In Section “Explanations for the NI Findings,” we examine the principal explanations for the NI findings and argue that two theories, the arousal hypothesis and the recent Association by Similarity Theory (AST), have better prospects than the others. In Section “Proposals for Experimental Design,” we investigate what experimental design can best provide evidence for differential imitation, focusing on how AST may be verified. In Section “Significance for the Field of Social Cognition,” we discuss the implications of NI for the field of social cognition. Overall, our goals are to help clarify the complex theoretical issues at stake and suggest fruitful guidelines for empirical research.

### The Operational Definition and the Importance of Keeping it in Mind

In order to operationalize imitation, Meltzoff and Moore (1977) postulated the following: infants imitate a particular behavior if there is a statistically significant increase of that behavior in the presence of the modeling of that behavior as compared to the modeling of alternative behavior(s). For instance, they tested whether “infants produce more tongue protrusions (TPs) after an adult demonstrates TP than after the same adult demonstrates mouth opening (MO), and vice versa” (Meltzoff and Moore, 1977, p. 76). Thus, NI was defined from the start as “differential imitation” (Meltzoff and Moore, 1977, p. 76) and that definition has been employed in the great majority of empirical studies concerning NI (Meltzoff, 2005; Coulon et al., 2013; Oostenbroek et al., 2016).

Hereafter we call the increase that a gesture exhibits when the corresponding model is presented compared to when other models are presented Comparative Increase for the Corresponding Model (CICM). In this first section, we focus on TP and MO as examples, because they are the actions for which CICM is most frequently reported. In particular, the CICM for TP is reliably documented (Heimann et al., 1989; Meltzoff and Moore, 1992; Ullstadius, 1998; Coulon et al., 2013; Nagy et al., 2013).

Why is imitation operationalized as differential imitation? The reason—already specified by Meltzoff and Moore (1977)—is that imitation has to be distinguished from a global arousal response. Observing that TP increases in response to the TP model as compared to when the infant is presented with a still face does not in itself indicate imitation. In such cases it would be more parsimonious to think that the baby is aroused by the presentation of TP and increases its TP production as a consequence of its state of arousal. Indeed, we could point to the now well-known data reported by Jones (1996, 2006), that perceiving flashing lights, dangling toys, and classical music increases TP in infants. In the absence of more compelling evidence for imitation, one could think that what causes the TP response in the case of TP presentation is merely the arousal state, which is provoked just as well by other arbitrary stimuli. The perception of characteristic features of TP would not play a determinant role in generating the matching response and so the matching response could not count as imitation. The operational definition of imitation must neutralize the arousal explanation in advance; otherwise there is no guarantee that minimal conditions for imitation are met.

Meltzoff and Moore’s (1977, 1997; Meltzoff, 2002, 2005) insistence on the claim that a plurality of gestures exhibits CICM indicates that the operational definition of imitation should be understood as entailing reference to such plurality of gestures. There is differential imitation only if more than one gesture exhibits a statistically significant increase when the same gesture is presented compared to when other gestures are presented, i.e., *only if more than one gesture exhibits CICM.* In this regard, it is noteworthy that advocates of the arousal explanation ground their hypothesis on the claim that only one gesture presents CICM. Hence these theorists seem to accept that if more than one gesture were to exhibit CICM the arousal hypothesis would be falsified or seriously undermined (Anisfeld, 2005; Jones, 2009; Ray and Heyes, 2011).

The arousal hypothesis is untenable in the presence of differential imitation. This hypothesis can explain CICM for one gesture if that gesture’s modeling is more arousing than other models. In particular, the TP model may elicit more TPs than other models in a set because it is the most arousing stimulus in that set. Yet, if another model (e.g., MO) also elicits the corresponding gesture more than the other models (including TP), the arousal explanation encounters a problem. Indeed, Meltzoff and Moore (1977; Meltzoff, 2002, 2005) understood arousal as a global state that increases overall action production, not just a particular action. Thus, if TP has been assumed to be the most arousing model to explain the CICM of TP, TP should solicit more MO than the MO model because it provokes a greater state of arousal and, among other responses, more MO. The same holds for the other gestures in the set under consideration. Therefore, according to the arousal hypothesis, TP would cause a comparative increase in other gestures as well, excluding the possibility that comparative increase for these other gestures is caused by their corresponding models. So understood, the arousal hypothesis predicts the absence of differential imitation for a plurality of gestures.

There is, however, a more promising way to elaborate the arousal hypothesis: Jones (2009) proposed that arousal might actually elicit just one particular behavior. Specifically, the TP model could cause an increase in TP, but not in MO. Furthermore, increase in TP could affect the frequency of spontaneous MO, since an infant engaged in TP production would have fewer occasions to produce MO (Ray and Heyes, 2011). This consideration could explain CICM for MO with respect to the TP model: MO production would be less impeded by TP in presence of the MO model. However, granted all this, the arousal hypothesis remains at a loss to explain the CICM for MO with respect to still face or models other than TP. If arousal does not increase MO, then the MO model should not elicit more MO than a still face. And how could arousal explain that the MO model solicits more MO than the lip protrusion (LP) or head rotation (HR) models? An *ad hoc* readjustment positing that MO is more arousing than these other models would be highly unsatisfactory (see Proposals for Experimental Design).

In short, if differential imitation is documented for more than one gesture, and data analysis entails more than the comparison between the effects of two models, the arousal hypothesis fails. Generally, the more gestures exhibit CICM the more impractical the arousal explanation becomes. Nobody would defend the idea of different kinds of arousal for differential imitation: an arousal caused by and causing TP, an arousal caused by and causing MO, and so forth.

In concluding this first section, we state two reasons why it is important that contributors to the debate keep the operational definition of imitation firmly in mind. First, the operational definition establishes the primary empirical question that should be investigated. Does differential imitation involving a plurality of gestures exist? If the answer is no, then arousal is the most sensible explanation and the debate is settled. If the answer is yes, then it is legitimate to assume that it is the perception of the specific action features of the model (not just its arousal value) that plays a role in generating CICMs.

Accordingly, the operational definition allows one to decide what findings may count as evidence for imitation. For example, Simpson et al. (2016) do not provide evidence for NI, although the authors use this label for their findings. Simpson et al. (2016) found that about half of the newborn monkeys under investigation produced a greater increase of lipsmacking from baseline (passive face) to the lipsmacking model than from baseline to a control model (a rotating colored disk). Lipsmacking is known to occur more often in presence of different types of arousing stimuli, just like TP in human infants (Subiaul, 2010). The very fact that in Simpson et al. (2016) lipsmacking increased in presence of the rotating colored disk suggests that this stimulus provoked some arousal. Thus, Simpson et al.’s (2016) results with newborn monkeys may be easily explained by assuming that half of the infants found the lipsmacking human model more arousing than the rotating disk. Ultimately, the term “imitation” is not warranted in this study because differential imitation is not attested for more than one gesture.

Second, the operational definition orients theories of NI because it specifies the actual phenomenon that needs to be explained. Given that imitation is defined as a differential increase in the frequencies of gestures that are spontaneously produced, it seems clear that theories of NI must relate imitative responses to spontaneous activity and must seek a satisfactory account for this relation. Indeed, all studies of NI investigate gestures spontaneously produced by infants. Even Meltzoff and Moore (1994), who investigated the “unusual” tongue-protrusion-to-the-side behavior, found that, in a 90-s test period, such behavior was spontaneously produced by 6 infants out of 30 who had never seen the tongue-protrusion-to-the-side model.

Furthermore, there is another feature in the operational definition of remarkable theoretical import. Consider the evidence for MO imitation reported in two key studies in the NI literature: Meltzoff and Moore (1977, 1994). The former study found a significant CICM for MO with respect to baseline and TP (Experiment 2). In the latter study, there was also CICM for MO with respect to baseline and TP, but it did not reach statistical significance. Evidence for MO imitation was provided by a significant increase in the duration of MO in response to the MO model compared to the control conditions—we may describe this evidence as a CICM for MO duration. However, the remarkable feature of MO imitation in both studies is that infants produced more TP than MO in response to the MO model. In Meltzoff and Moore (1994), the sum of the mean frequencies of TP in response to MO (31.90) was well over twice the corresponding value for MO (13.70). Hence, an imaginary observer attending only the trials in which MO was presented would have seen infants performing more than two TP for every MO they produced. This is not what we would expect based on the prototypical idea of imitation, according to which it is natural to think that infants imitate MO if they produce more MO than TP in response to the MO model. Indeed, it is not unlikely that, if unaware of the hypothesis under investigation and without the possibility of comparing her observations with the control conditions, the imaginary observer would have not described the interactions she attended as MO imitation. Yet these findings count as MO imitation because they meet the requirements of the operational definition—Meltzoff and Moore (1997, 1994) found CICM for TP and MO. Therefore, the operational definition does not guarantee that imitative responses appear as such to an observer. Differential imitation may not look like ordinary imitation!

It follows that a theory of NI may be misguided if tends to describe NI as being similar to ordinary imitation. Moreover, hypotheses that base the social function of imitation on its recognizable appearance should be treated with great caution. Nonetheless, it would be wrong to reject the term “imitation” altogether just because responses may not be visible as ordinary imitation. As already mentioned, the operational definition of imitation guarantees a minimal requirement for imitation: infants must perceive characteristic features of the modeled action and then they must tend to produce the same action on the basis of their perception of its characteristic features, not because of other irrelevant properties of the perceived model (e.g., its arousing properties).^2^ In absence of a better word, all phenomena presenting this minimal kind of perception-action connection can be labeled as “imitation,” granted that not all of them will exhibit the features of prototypical imitation.

### Explanations for the NI Findings

There are two main types of explanations for the NI findings, depending on what goal they set out to achieve, i.e., on what they take the *explanandum* to be. There are theories for which the only thing that has to be explained is the CICM for TP (and, perhaps, the accidental appearance of CICM in other rare cases). Other theories aim at explaining differential imitation. So, on one side, there are theories that assume that findings do not and will not prove the existence of differential imitation. On the other side, there are theories that rely on the opposite assumption. In this section, we review principal theories on both sides and examine which ones have the most explanatory power.

Before we start our critical survey, we need to make it clear that our discussion is guided by our overall appraisal of the extant findings. We think that detractors of differential imitation have done a good job in showing that existing evidence for differential imitation is not compelling (Anisfeld, 2005; Jones, 2009; Ray and Heyes, 2011; Heyes, 2016). However, we do not think this justifies denying its existence. It is possible that current findings point to a nucleus of differential imitation the existence of which will be firmly demonstrated by future research. In our view, the extant empirical literature, despite its extensiveness, is still ambiguous.

Let us then begin with theories based on the empirical claim that *only* TP exhibits CICM (i.e., differential imitation does not exist). Anisfeld (1991) presents two theories of this kind: the Innate Releasing Mechanism (IRM) and the “attention, response release” hypotheses. IRM (Jacobson, 1979; Abravanel and Sigafoos, 1984; Bjorklund, 1987) posits that TP is a fixed action pattern released under a relatively specific set of stimulus conditions (e.g., stimuli, including the TP model, resembling an approaching nipple). An IRM is more flexible than a standard reflex (e.g., Moro reflex); hence the notion seemed to account for the documented variability of the TP behavior. The second theory, the “attention, response release” hypothesis simply supposes that infants inhibit spontaneous TP when their attention is captured by the TP model and then discharge a higher rate of TP when the model disappears, as a function of the energy that has built up internally during inhibition. This latter proposal can be considered an arousal explanation. Indeed, one can assume that arousal is precisely what builds up during model presentation and is expressed as higher rates of TP in the model-free response period.

#### Arousal

Since Jones (1996, 2006) demonstrated TP increases in response to a wider set of stimuli than what the IRM hypothesis predicted, recent skeptics of differential imitation converged on the arousal explanation for the CICM of TP (Anisfeld, 2005; Jones, 2009; Ray and Heyes, 2011). We recapitulate five main reasons for why this is a viable explanation:

(i) Differential imitation is disputable because findings are highly variable and often negative (e.g., Oostenbroek et al., 2016).
(ii) A variety of arousing stimuli in different modalities elicit TP (Jones, 2009).
(iii) Jones (1996) found that 4-week-olds looked longer at a TP display than a MO display, confirming the assumption that TP is more arousing to infants than other modeled actions. This assumption allows the arousal hypothesis to account for reliable CICM for TP.
(iv) Tongue protrusion behaves like other spontaneous stereotypies characterizing development which increase as a result of non-specific stimuli connected to arousal (Keven and Akins, 2016).
(v) Jones (1996, 2006) found that arousing stimuli other than modeled actions (lights, toys, music) cause a specific increase of TP, but not a diffuse increase of other actions as well. Thus, the arousal hypothesis can explain the fact that the TP model does not produce a comparative increase in the production of other gestures (e.g., MO).

Some (e.g., Simpson et al., 2014) would not grant the first point, that the existence of differential imitation is disputable. In any case, the other four points are so compelling that even defenders of differential imitation should accommodate arousal as a factor contributing to the CICM for TP. Arousing stimuli of disparate kinds elicit TP and the TP model is an arousing stimulus. At a minimum, imitation defenders should accept that in addition to being imitative, TP is *also* an arousal response.

We now move to accounts that aim at explaining differential imitation.

#### Genetically Programmed Direct Matching (GPDM)

Genetically programmed direct matching is the name we assign to the psychological model of differential imitation that is most naturally associated with the classical genetic account of mirror neurons (Cook et al., 2014; Tramacere et al., 2015). Jones (2009) introduces this type of model as requiring infants to have significantly fewer cognitive abilities than what AIM assumes. In fact, GPDM heavily relies on an evolutionary story: populations in which newborn brains were able to automatically connect the perception of specific actions with their corresponding action plans were selected. This is why we qualify this matching of action perceptions with action plans as “genetically programmed.”

That action plan activation is automatic means that the infant does not need to know why it has an impulse to act in a certain way rather than another. *The infant perceives a specific action and then has a tendency to act in a specific way.* According to GPDM, this is a complete description of the psychological states underlying imitation. Regulating which action tendency follows which perception is the work of neural mechanisms selected through evolution, but no psychological operation of the infant is directed at these mechanisms. In other words, it is not necessary for infants to recognize equivalences between modeled and executed actions. NI may still serve a social function in that it affects caregivers and positive infant-caregiver interaction is promoted (Heimann, 2002).

We note that GPDM as a model of differential imitation has not been developed to account for the details of the empirical literature. Moreover, it has not been defended in opposition to the most well known model of differential imitation (AIM). Here, we consider it only in so far as alternative explanations cannot be understood if it is not clear how they differ from GPDM. Hence, in this subsection, we anticipate its differences from other theories, i.e., AIM and AST.

We qualify perception-action matching in GPDM as “direct” precisely because it does not require infants to recognize self-other similarities. This is a critical difference from Meltzoff and Moore’s (1997) AIM model. Meltzoff and Decety (2003, p. 494) distinguish the mirror neuron based model from AIM because the latter posits “an active comparison and lack of confusion between self and other.” Indeed, Meltzoff and Decety propose that mirror neurons may not be sufficient to implement the psychological operations necessary for imitation. Something more (the inferior parietal lobe) is likely to be required to implement the recognition of “both the similarity and the distinction between actions of the self and other” (Meltzoff and Decety, 2003, p. 494). The point is repeated in Meltzoff (2009, p. 38) where it is suggested that mirror neurons are not well suited to account for the psychological phenomena AIM seeks to explain, notably “response correction” and “the imitation of novel acts.”

**FIGURE 1:**
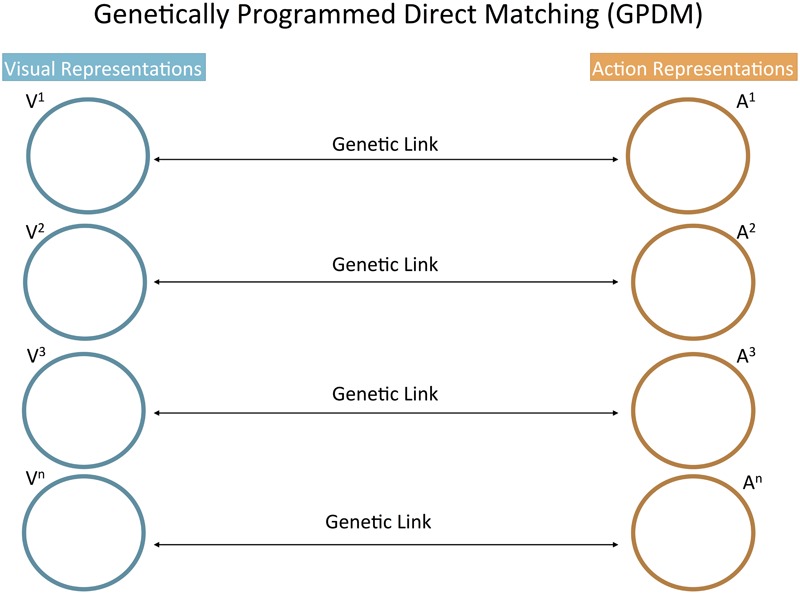
Conceptual schematic of the GPDM model. Visual representations (Vn) are connected with the corresponding action representations (An) through automatic connections encoded by the genes. These genetic links are not domain-general processes of association; rather, they were specifically selected for social or socio-cognitive functions. This schematic is comparable in format with those of AIM and AST in **Figure 2**. In both **Figures 1, 2**, an “action representation” refers to a spontaneous habitual action. Accordingly, an action representation includes the representation of the characteristic features of the action as experienced proprioceptively and, therefore, the representation of those characteristic features as integrated in the “action plan” for the action.

In order to anticipate its difference from AST, we need to emphasize that GPDM does not assign any functional role to the domain-general process of association by similarity. Perception-action connections are essentially different from the ordinary process by which a current visual stimulus is interpreted in light of a similar perceptual experience (generalization). Rather, in GPDM, perceptual representations are connected with corresponding action representations through genetic links that were specifically selected for social or socio-cognitive functions (**Figure 1**).^3^

#### Active Intermodal Matching (AIM)

The “crux” of the AIM hypothesis is that imitation is “intentional” or “goal-directed;” the goal is to achieve a “match” between perceived and executed actions.^4^ Goal-directedness is what allows Meltzoff (2010, p. 19) to define NI as “genuine imitation.” Indeed, true imitation is traditionally described as having an active, intentional character (Piaget, 1951/1962). Evidence for goal-directedness is a presumed process of response correction in which infants use the target/goal as their criterion for correction (Meltzoff and Moore, 1997).

Imitation is achieved through a “comparison” computation described in Meltzoff and Moore (1997, pp. 185–186). The comparison has two inputs: one is visually perceived action features and the other is proprioceptively experienced action features. Specifically, the computation compares the “configural relation between organs” and/or the “speed, duration, and manner” of the modeled action with the configural organ relations and/or dynamic properties of the infants’ own actions (Meltzoff and Moore, 1997, p. 184, p. 189). If the two inputs are dissimilar, the output is a “mismatch,” and then the infant executes a new attempt. If the two inputs are similar, the output is a “match” and the infant has recognized the similarity between her own and the other’s actions. For this reason, imitation entails the recognition experience or the basic perception: “That seen event is like this felt event” or “Here is something like me” (Meltzoff, 2002, 2005, 2007, 2010). Note that it is essential to AIM that the two inputs entering the computation are clearly separate, as shown in Meltzoff and Moore’s (1997 p. 186) schematic of AIM. If there are not two distinct inputs, the idea of a comparison for the detection of similarities and dissimilarities falls apart. Hence, Meltzoff and Moore (1997) insist that the visual representations of the modeled actions must be “independent” or “separate” from the corresponding proprioceptive representations.^5^

In order to make the “action system” commensurable with the “perceptual system,” evolution provided infants with a “supramodal representational system” (Meltzoff and Moore, 1997, p. 186). This system provides “the lingua franca, the abstract code, for connecting self and other” (Meltzoff, 2010, p. 16). Nature designed infants with an “imitative brain” through “Darwinian means” (Meltzoff, 2005, p. 55 and p. 57). This “special neural-cognitive machine” evolved to ground social cognition, including theory of mind and testing others’ identity (Meltzoff, 2002, p. 9; Meltzoff, 2013).

One of the main reasons for being skeptical of AIM is the following: if infants have a developmentally crucial “innate propensity to imitate” for which nature evolved a specialized comparison mechanism (Meltzoff, 2002, 2007; p. 7), then the empirical literature on differential imitation would be more robust than it currently is (point ii. below). An extensive critique of AIM can be found in Vincini and Jhang (unpublished). Here we list some of our objections.

(i) Progressive match between infants’ responses and the model does not have to be understood as response correction. Rather, the phenomenon is more parsimoniously interpreted as “increase in vigor [and amplitude] with response repetition, or […] perceptual learning—[…] the formation of a better perceptual representation of the modeled movement with repeated exposures” (Ray and Heyes, 2011, p. 96). In plain words, there is no clear evidence for the crux of the AIM hypothesis!
(ii) If infants have an innate propensity to imitate and NI is foundational for social cognition, infants should imitate often and imitation should be easily detectable, but results are highly variable and often negative (Ray and Heyes, 2011).
(iii) If neonatal propensity to imitate fulfills a central socio-cognitive function, there seems to be no good explanation of why imitation appears more easily in laboratory settings (Meltzoff and Moore, 1992, 1994; Coulon et al., 2013) than in naturalistic environments (cf. Oostenbroek et al., 2013, p. 337; Oostenbroek et al., 2016).^6^
(iv) A comparison mechanism is particularly appropriate if there is a wide range of gestures that potentially count as “matches” for their respective models, since the system needs to discriminate the matching response from the large set of non-matching responses. However, current findings suggest a quite limited range of candidate gestures for differential imitation (Ray and Heyes, 2011).
(v) If the infants’ goal is to match the target they are presented with, infants should imitate different gestures equally. Nevertheless, two of the gestures listed by Meltzoff and Moore (1997), i.e. TP and MO, exhibit CICM more often than the others, and TP more than MO.^7^ AIM cannot explain this fact without resorting to auxiliary assumptions.
(vi) Meltzoff and Moore (1992) explain the “drop out” of imitation at 2–3 months by assuming that infants become more interested in other forms of interactions. Others have offered explanations more clearly grounded in motor development (Keven and Akins, 2016). In the third post-natal month, infants engage the social environment more actively than before (Rochat and Striano, 1999), so why does an active social behavior as that described by AIM decrease? Importantly, Meltzoff and Moore’s (1992) explanation does not account for the correlation between the declining rate of TP and MO imitation and the declining rate of TP and MO spontaneous production (Ray and Heyes, 2011).
(vii) Active intermodal matching seems to promote an inflationary reading of the findings. For example, Meltzoff (2005, 2010 p. 71; pp. 18–19) describes the Meltzoff and Moore’s (1977, 1994) findings as if imitation simply implied responding to a model with the corresponding action; furthermore, Meltzoff and Moore’s (1997, p. 182) depict their 1994 results on tongue-protrusion-to-the-side imitation as the “generation of novel behaviors not found in baseline activity.” However, in Section “The Operational Definition and the Importance of Keeping it in Mind,” we noted (a) that MO imitation was not affected by the fact that infants responded with more TP than MO to the MO model, and (b) that tongue-protrusion-to-the-side did appear in baseline. Therefore, interpreting differential imitation as “genuine imitation” may mislead one to characterize it as unduly similar to prototype or culture-learning imitation.

#### Association by Similarity Theory (AST)

Although anticipated by Kinsbourne (2002), AST was recently introduced as a detailed alternative model to AIM by Vincini and Jhang (unpublished). AST relies on domain-general processes of association by similarity and can be considered as a consistent application of Prinz’s (2005) ideomotor theory to NI. AST interprets NI as differential induction of spontaneous behavior through similarity.

Association by similarity is one of the main principles in traditional associationism (Hume, 2000), has been recognized as fundamental to a number of psychological phenomena (Larkey and Markman, 2005; Allen, 2012), and studied in sophisticated ways (Tversky, 1988; Nosofsky, 1992; Vigo, 2009). Considering that practically any organism capable of learning must be able to generalize its behavior to stimuli similar to those already encountered, it can be supposed that similarity was functional very early in evolution (Shepard, 1987).

From a phenomenological perspective, association by similarity is a form of “operative intentionality” (Merleau-Ponty, 2012), indicating regulated ways in which experiences are connected so as to make our coherent experience of the world, and any further higher-order cognition possible. In general, association by similarity designates the process by which a present experience or cognitive event is connected to a similar past experience, reactivating or re-enacting the content of the past experience. For example, I am used to responding to specific stimuli in a specific way; thus, when I encounter a new similar stimulus, a specific motor response is facilitated (Dreyfus and Dreyfus, 1999). From a cognitivist perspective, similarity can be defined as the process by which bits of information tend to activate wholes in which they are normally integrated. For instance, in Hebbian learning models of perception, bits of information that are activated together become associated to constitute a complex object representation. Hence, a novel activation of an information bit due to sensory input facilitates the activation of the associated bits that complete the representation of the object, making object recognition possible (Mongillo, 2012).^8^

In developmental studies, the habituation procedure relies precisely on the relation of similarity between present and past (harmless) stimuli (Van Heteren et al., 2000; Sommerville and Woodward, 2005). Neonates show preference for familiar stimuli, i.e., stimuli similar to experiences had before birth, in the domains of audition, taste, and smell (Hepper, 2015). Importantly, similarity is context-dependent and, in each case, it must be specified what features of the stimulus are relevant to the (action-oriented) experience of the subject (Decock and Douven, 2011). In the case of modeled actions in NI studies, the features of the stimulus that may be relevant to the functioning of similarity are action features that are habitually instantiated in the infant’s own behavior. Specifically, each model will present features that are routinely experienced in one of the infant’s behavioral stereotypies.

**FIGURE 2:**
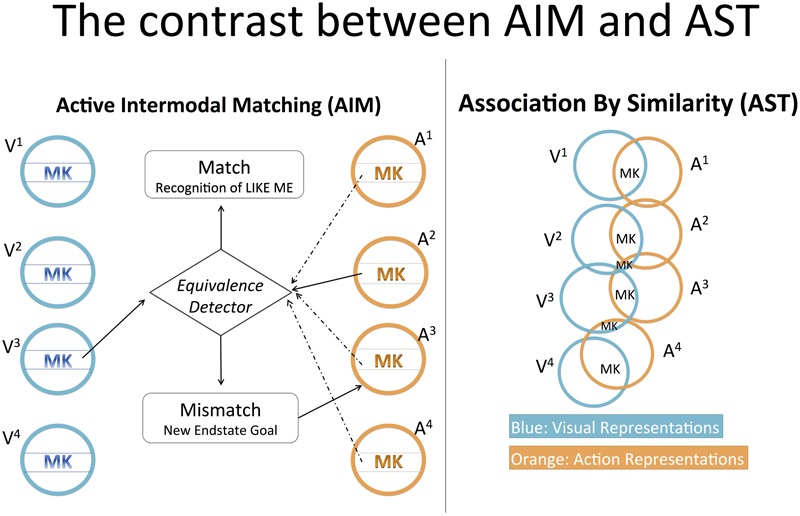
The contrast between AIM and AST. AIM postulates a comparison between visual and action representations. If the comparison gives a “mismatch” output (e.g., A2 does not match with V3, judged by the Equivalence Detector), a new action representation is activated for a novel imitative attempt. If the comparison gives the “match” output, the infant has recognized a self-other similarity. In AST, there is no such comparison. Each visual representation overlaps the most with the corresponding action representation (i.e., V1 overlaps the most with A1 and so on); thus, when a visual representation is activated, the corresponding action representation tends to be activated too. The action’s “morphokinesis” (MK) designates the set of action features experienced both in visual perception and action execution; the set includes the action’s kinetic features and the peculiar configurations of body parts achieved through the action. In AIM, the peculiar MK of an action constitutes two distinct inputs that enter the comparison computation. In AST it simply indicates the information that visual and corresponding action representations share in common.

The contrast between AST and AIM (**Figure 2**) can be discussed by examining four crucial differences between AIM and Prinz’s (2005) ideomotor theory, of which AST is an application. First, as seen in Meltzoff and Moore’s (1997, p. 180 and p. 186) diagrams, AIM posits no overlap between the perception and action systems; rather, it posits a “supramodal” system specifically evolved to make perception and action commensurable. In contrast, Prinz’s more parsimonious scheme (1997, p. 130) shows intrinsic overlap between perception and action. There is no perception of an action without the representation of its dynamic spatiotemporal features or of the final state achieved through it; in the same way, there is no action planning without representation of the action’s dynamic features and final state. Prinz’s (1997, 2005, p. 144) hypothesis is that the very same resources employed in perception to represent specific action features are employed in action planning to represent the same action features. The strength by which a specific perception induces a specific action depends “on the degree of similarity, or [representational] overlap” between them.

Second, in AIM, the imitative response is actively determined by the infant, who modifies its responses in light of its goal. In contrast, in the ideomotor theory of imitation, action facilitation is determined passively by perceptual experience. In Prinz’s texts (Prinz, 1990, 1997, 2002, 2005; Massen and Prinz, 2009; Prinz et al., 2009), perceptions “induce,” “modulate,” “suggest,” “facilitate,” “awaken,” “elicit,” or “prime,” corresponding actions. The perceptual system may even be said to “seduce” the action system. All these expressions denote passivity. Indeed, according to the ideomotor principle, if the “idea” of an action comes to mind it will tend to bring about the action (actually bringing it about if antagonistic tendencies are inhibited). Now, the “idea” of an action, i.e., its “representation” or “action plan,” can be awakened by perceiving the action. Thus, insofar as perception evokes the representation of an action, perception tends to induce that action. For this reason, Prinz (2002, p. 160) states: “action imitation is therefore a natural by-product of action perception.” Certainly, passive induction presupposes activity, both in general ideomotor theory (which assumes a motor repertoire sufficiently exercised) and AST (according to which NI experiments take advantage of infant spontaneous and habitual action execution). Nonetheless, which action is facilitated over others is a passive effect of perception. In short, while the crux of the AIM hypothesis is “the active nature of the matching process” (Meltzoff and Moore, 1997, p. 182), ideomotor theory and AST propose *passive similarity-based induction*.

Third, as noted above, it is essential to AIM that representations of visually specified and proprioceptively specified action features be two “separate” inputs of the comparison computation. In contrast, Prinz’s (2002, p. 153) claim is that “*identical* representational structures are involved in the perceiving and the performing of actions” (our emphasis) and that, when these structures are activated in perception, they tend to awaken the action plans in which they are habitually integrated. No comparison computation is required for imitation. As a consequence, there is no psychological act having similarity or dissimilarity as its content (“that seen event is like this felt event”). For ideomotor theory, “recognition of similarity” is not necessary for imitation. If it were, action imitation would not be “the natural by-product of action perception,” but rather the achievement of a mechanism to be added to perception, i.e., the mechanism for comparison/similarity recognition. Obviously, ideomotor theory assumes a “functional role” of similarity (Prinz, 2002), but this does not imply positing that similarity is the object of a mental act. Similarity merely regulates how specific perceptions facilitate specific actions.

Fourth, for AIM, imitation is underlain by a specialized mechanism specifically evolved for social and socio-cognitive functions.^9^ In contrast, Prinz (1997) provides evidence for the functioning of similarity in simple perception-action tasks outside the social domain. Hence, Massen and Prinz (2009, p. 2357) insist that “imitation is not based upon special purpose mechanisms, but, rather, relies on the general organization of learning and action control.”^10^

Association by similarity theory consistently applies these four features of ideomotor theory to NI. It goes further than ideomotor theory in that it brings to the fore the distinction between association as mere reactivation of sensory-motor wholes and the recognition experience of the form “a is like b” emerging from the comparison between distinct representations. It also provides the necessary assumptions to explain the details of NI. Hence AST emphasizes that each infant’s behavioral habit or stereotypy instantiates action characteristics experienced proprioceptively (i.e., a specific pattern of movement, of configuring body parts across time).^11^ When a corresponding model is presented, infants experience the same action characteristics visually. AST hypothesizes that the same resources that represent those action features in action planning and execution represent those features in perception. Therefore, when those resources are activated in perception, they tend to activate the specific motor resources in which they are habitually integrated during action planning and execution. In this way, a specific action plan, i.e., an action tendency or habitual action possibility, is awakened by the perception of a particular model. Hence model presentation contributes to promoting a specific action tendency over other spontaneous tendencies. Importantly, it does not suffice that the visual stimulus resembles the proprioceptively experienced action pattern in some rough way. The stimulus must be capable of keeping the infant calm and attentive; therefore, it must fit the innate, but rapidly developing perceptual preferences of human newborns (Simion et al., 2011). This explains why non-social stimuli are often not capable of inducing imitation (Jacobson, 1979; Abravanel and DeYong, 1991; Legerstee, 1991; Oostenbroek et al., 2016).

Association by similarity theory does not posit that infants have an innate propensity to imitate or that NI is foundational for social cognition. For AST, model presentation in the experimental setting is simply an artificial (non-ecological) attempt at inducing a habitual action. The action-awakening effect of the model is a relatively weak factor compared to the highly variable affective states and action tendencies that otherwise condition infants. AST thereby accounts for the high variability of the extant findings: because imitation is not supported by a specialized propensity, alternative impulses also determine infant responses. If infants at this age do not care about matching what others do, but merely comply with their action tendencies (whether spontaneous or partially induced), NI will not present the empirical consistency one could expect on the basis of AIM, but rather a messier and even conflicting panorama of results, much like what the extant literature shows.

We propose that AST is more plausible than AIM (Vincini and Jhang, unpublished). Arguments in favor of AST correspond to arguments against AIM. Thus, the reader may compare the following list with the one provided in the previous subsection.

(i) In AST, progressive match between response and model is parsimoniously understood as increase in vigor and amplitude of an induced response through energizing specific bodily parts, response initiation, and repetition. This Piagetian idea (that action calls for, or encourages, its repetition) is consistent with the observation that response rates increase over the course of the experiment (Anisfeld et al., 2001; Anisfeld, 2005). Furthermore, given the immaturity of the visual system, it should not be taken for granted that infants immediately perceive the relevant features of the model (Jones, 2009). Repeated exposure allows infants to see the model’s action features more distinctively; hence induction should occur in later phases of the experiment when action features are effectively perceived.
(ii) Association by similarity theory explains the ambiguity in the extant empirical literature. Given the lack of any “innate propensity to imitate,” complex variables and random factors will often prevail in the infant psychological space and induction will often not be successful.
(iii) The fact that imitation appears more frequently in laboratory settings than naturalistic environments is elegantly explained. NI is not an evolved response for social interaction, but, in such experimental settings, an artificially induced one. In contrast to environments rich in potential distractors, imitation occurs more easily in settings where conditions (e.g., uniform background, model luminance, temperature) maximize a calm and attentive attitude in the infant, which is usually rather fragile.
(iv) Association by similarity theory accommodates evidence suggesting that the range of imitated gesture is not wide. Indeed, it is plausible that similarity-based induction may occur only for one or two gestures whose action features are well differentiated in visual and proprioceptive experience (e.g., a gesture frequently executed and whose action features are markedly different from those of other gestures).
(v) It is a core assumption in AST that there will be correlation between imitation and spontaneous production: the more habitual the gesture, the more easily it will be induced. This prediction is confirmed. In Section “Active Intermodal Matching,” we noted that TP is the most imitated gesture and MO the second one. Here we add that TP is the gesture that occurs most frequently in spontaneous behavior and MO is the second most frequent.^12^
(vi) Association by similarity theory satisfactorily explains why imitation drops out at 2–3 months. Because AST understands imitation as differential solicitation of actions that already tend to occur spontaneously, it predicts that if the actions in question stop being spontaneously executed, it will be difficult if not impossible to solicit them. This prediction is verified by the existence of a correlation between decrease in imitation and decrease in spontaneous execution (Keven and Akins, 2016). Additionally, AST sheds light on another factor that may contribute to imitation drop out. When infants become more active in social interaction at 2 months (Rochat and Striano, 1999), they will be less disposed to let the “choice” of their behavior be determined passively by a stimulus. Infants will behave according to a self-determined stance more often and, consequently, actions passively induced by model presentation will be less frequent.
(vii) Association by similarity theory does not require differential imitation to be recognizable as ordinary imitation, because experiment-based induction is an artificial effect and may be detectable only in comparative analyses.

### Proposals for Experimental Design

The preceding discussion has left us with two hypotheses that are more promising than the others: arousal and AST. Arousal, as an explanation of NI, is essentially a negative hypothesis in the sense that negative findings concerning differential imitation are the primary evidence in its support. Indeed, we can say that arousal is best verified by falsifying positive imitation hypotheses. As viable imitation hypotheses are disqualified by the findings, arousal emerges as the most sensible explanation. Hence, in this section, we focus on procedures to test AST as a positive hypothesis for NI.

Remember that the primary empirical question is whether differential imitation exists and, for this reason, our discussion aims at identifying procedures for better addressing this question. However, one should also keep in mind that different models of differential imitation imply different experimental procedures to test their validity. Specifically, from AIM derive experimental procedures that have been consistently implemented in Oostenbroek et al. (2016). AST predicts that those procedures will likely *not* be effective in detecting or inducing imitation and recommends opposite procedures. Thus, before outlining the procedures to test AST, it is opportune to recall those deriving from AIM.

Oostenbroek et al. (2016) designed an experimental procedure to test the hypothesis that imitation has the kinds of socio-cognitive functions postulated by AIM. If infants have an innate propensity to imitate and this propensity is foundational for social cognition, if imitation is a behavior characteristic of “typical newborns” (Meltzoff, 2010, p. 16):

(i) Results should be generalizable to typical newborns; so imitation should be tested in large populations; Oostenbroek et al. (2016) examined 106 infants.
(ii) Imitation should be detectable through measures of typical behavior, e.g., by averaging rates of action production across all infants, as Oostenbroek et al. (2016) did.
(iii) The increase of production of the modeled action should be significant with respect to any control model taken of a large set of different kinds of models; Oostenbroek et al. (2016) used 11 models, including facial, non-social, vocal, and hand gestures.
(iv) Infants should change their behavior as the behavior to be matched changes; thus, imitation of different models should occur in the same test session; Oostenbroek et al. (2016) presented 11 models in a row in an 11 min long session.
(v) Imitation should occur in naturalistic environments; Oostenbroek et al. (2016) tested infants at their homes.

Because they found no imitation, Oostenbroek et al. (2016, p. 3) concluded AIM is “not empirically supported and should be modified or abandoned altogether.”

Assuming AST, we can explain why a design of this kind would not work and suggest alternative procedures:

(i) Testing a very large sample makes it impractical for experimenters to focus on finding the optimal conditions for induction in each infant. For example, Oostenbroek et al. (2016) say little or nothing about how experimenters captured the infant’s optimal alert state and facilitated attention to the model. Experimenters should have the possibility to monitor the infant beginning 10–15 min after feeding and wait till the quiet alert state comes—State 4 in Brazelton and Nugent’s (2011) scale. The infant must be calm, content, and attentive as much as possible for the entire session. There should be a preliminary phase in which the experimenter seeks the optimal posture for the infant and attracts attention to the model (e.g., producing sounds without opening the mouth).^13^ Simpson et al. (2014) proposed a sample size of at least 26 infants based on *a priori* power analysis. Because experimenters should focus on creating favorable affective conditions, we propose sample size need not be greater than 30 infants.
(ii) Imitation as differential induction does not occur often; so averaging frequencies across infants who have all sorts of different action tendencies tends to “iron out” genuine episodes of imitation. Infants are not unified by a propensity to imitate; therefore, each infant should be treated independently. We propose each infant be its own control (i.e., comparing responses to different models for each infant).
(iii) Increasing the number of control models increases the possibility that random factors in action production will obscure imitation effects. Moreover, different kinds of model may interest or arouse infants to different extents, but interest/arousal levels should be kept as constant as possible across models. We propose using no more than four facial models.
(iv) Infants are not imitating machines and cannot be expected to imitate 11 models in one session.^14^ An induced action can be executed more than a minute after model presentation (see Heimann, 2000 for a discussion of the slowness of infant responses). In Oostenbroek et al.’s (2016) design, “slow” responses of this kind may end up counting against the imitation effect (because they can occur in the response period for a subsequent model). We propose there should be only one model in each session and response phases should be longer.
(v) Naturalistic environments are rich in potential distractors and do not permit optimized conditions. Sessions should occur in silent laboratory settings with adjustable temperature and lighting. The visual background should be a uniform soothing color and the modeler’s face must be spotlighted and its luminance regulated. Note that it is not a problem to include in the experimental design an unusual face presentation such as the modeler’s spotlighted face. Unlike AIM, AST does not posit that NI is a typical behavior occurring in the ordinary conditions of infant-caregiver interaction. Quite the contrary, AST recommends making use of conditions, whether usual or unusual, that may enhance attention to the features of the modeled actions. Spotlighting the modeler’s face may well be one of those conditions, given that NI studies implementing it have produced positive results (e.g., Meltzoff and Moore, 1989, 1994; Legerstee, 1991; see Kugiumutzakis, 1999, for a similar procedure).

Experimental procedures in the extant empirical literature vary widely (Simpson et al., 2014). Some studies already apply the procedures we propose to different extents (e.g., Heimann and Schaller, 1985; Meltzoff and Moore, 1992, 1994; Kugiumutzakis, 1999). Therefore, our goal is not to outline innovative designs, but to specify procedures that can best detect differential induction (i.e., test AST). Our proposals are somewhat abstract and ideal, but may guide experimentalists in making concrete choices. The first proposal below can be classified as a “Many-Models” design because each infant sees more than one model in the course of the experiment.

The experimenter monitors the infant and takes it to the laboratory room when it is ready. The session begins with a 120-s acclimatization phase in which the experimenter finds a comfortable position for the infant and leaves it to acclimate to the environment. When the acclimatization phase ends, the experimenter’s face is spotlighted and an attention-catching phase begins in which the experimenter makes sure the infant is looking at her. The experimenter keeps the mouth closed and does not rotate the head up to this point. When the experimenter judges that the infant is looking at her, the baseline condition begins. This condition consists of 180-s uninterrupted passive face (PF) presentation. Subsequently, a new attention-catching phase occurs; when the attention requirement is fulfilled, the test phase begins. In total, the test phase is 180-s long. It is composed of four 15-s presentation phases (here we follow the suggestion from Simpson et al. (2014) that total presentation should be at least 60-s) alternated with four 15-s response phases in which the experimenter shows PF, plus an additional 60-s response period to allow for slow or delayed responses. During each presentation phase, a gesture is modeled four times. The next session occurs the following day. In total, there can be a minimum of 3 days-3 sessions and maximum of 4 days-4 sessions. Each session presents a different model and the order of models across days is counterbalanced. We propose the following models: TP, MO, LP, and HR. To track the longitudinal aspect of spontaneous activity and the development of action repertoires, each infant goes through all 3 or 4 sessions in the first, third, fifth, and seventh post-natal week.

This design allows experimenters to collect a fairly large amount of data and realize different kinds of analyses. In order to catch all possible imitative responses, it is best to collect gesture frequencies during response periods and presentation periods as well (Simpson et al., 2014). For each infant, the frequency of a gesture in response to the corresponding model must be compared to its frequency in response to other models and baseline. For each gesture, the analysis will divide infants in three groups: (1) highest frequency in response to corresponding model; (2) highest frequency in response to another model or baseline; (3) equal highest frequency in response to corresponding model and one other model or baseline. Under the null hypothesis (differential imitation does not occur), a certain distribution of the findings will be expected. For example, assuming there is a baseline condition and four models conditions, and that group (3) is empty, approximately a fifth of the infants should appear in (1). However, if (1) has a significantly larger proportion of infants, the imitation hypothesis for the gesture is verified.

In AST, we do not assume the existence of an innate imitation propensity enduring for the first 8 post-natal weeks. Moreover, levels of spontaneous activity may vary across weeks. Thus, longitudinal analyses averaging data from different weeks should be avoided and each week of testing should be analyzed separately. Obviously, when analyzing the data from each single week, it does not hurt to calculate the averages of a gesture across infants and compare them (as most published studies do), but we are more hopeful toward taking each infant as its own control. Among other things, this method of analysis allows one to examine particularly high rates of a certain gesture in single sessions (“outliers”). For example, suppose that two subjects perform a particularly high rate of MO in a single session. If the session in which this high rate appears is a MO model session, then it is likely that MO has been induced in these cases.

The five experimental conditions proposed acquire a special significance in AST. Study of spontaneous behaviors during baseline (PF) is key in AST because it allows one to test the assumption that the more a gesture is habitual, the easier it will be to induce it. If infants in group (1) present a higher baseline than the other groups for the gesture they imitate, AST is supported. The imitation-spontaneous production correlation can be investigated at an even more strictly individual level: if baseline production for a gesture is particularly high (compared to other gestures of the same infant or to the mean baseline for all infants), according to AST it is more likely that the infant will imitate that gesture.^15^

Tongue protrusion does not serve the purpose of verifying AST as well as other gestures. The arousal theorist can always interpret CICM for TP as an arousal response. Hence, if one of the four gesture/models has to be eliminated, we suggest eliminating TP. The interesting question related to TP is whether MO decreases with respect to baseline in response to TP presentation. If yes, the arousal theorist can rely on the argument that competition between TP and MO production explains CICM for MO with respect to the TP model. If no, the arousal theorist cannot rely on that argument. MO is a promising gesture for AST because we can assume that the proprioceptive experience of MO is relatively well differentiated from the experience of other actions. The proprioceptive experience of MO implies a morphokinetic rhythm alternating an expansion/stretching phase and a shrinking/relaxation phase; the same pattern can be perceived in the MO model. Thus, it should be possible to induce MO. It can be granted that the CICM for TP is an arousal effect, but if CICM for MO is proved to exist with respect to baseline and models other than TP, AST is supported. Meltzoff and Moore (1992, 1994) use CICM in MO duration as evidence for imitation. We agree that duration can count as an indicator of differential imitation because action induction may cause the action to be more pronounced and, therefore, longer than in spontaneous execution.

Head rotation is another action that is relatively well differentiated in proprioceptive experience because it presents a peculiar trajectory and rhythm (e.g., it is a more continuous movement than MO). However, HR imitation should be carefully distinguished from “perceptual tethering,” as Meltzoff and Moore (1989) ingeniously do. Most probably, LP is not well differentiated in proprioceptive behavior; thus, LP imitation should be rare or inexistent. This does not falsify AST because, for AST to be supported, it can be sufficient that a gesture other than TP present a consistent CICM.

Association by similarity theory can be tested through a “One-Model” design as well. We employ this expression to designate a procedure followed in Meltzoff and Moore (1994). Each infant is assigned to a group and each group is associated with a particular condition (one of the four models or baseline). Each infant is tested only under the condition associated with its group. In other words, the infant sees only the stimulus associated with its group in all sessions for 3–4 days. If the mean frequency for the action presented to a group is greater in that group than in groups to which other models or PF have been presented, the imitation hypothesis is confirmed. The advantage of the One-Model procedure is that, through insistence on one model over different sessions, induction may be facilitated. Indeed, Meltzoff and Moore (1994) found a positive result. The disadvantage is that it is not possible to take each infant as its own control.

Finally, we recommend that, apart from the change of model, experimenters seek to keep the components of the visual stimulus constant across all sessions.^16^ In all cases, eye-tracking should be used as much as possible to measure infant attention and determine what models are most interesting to infants (Coulon et al., 2013). Physiological measures can be used to register other aspects of infant arousal.

### Significance for the Field of Social Cognition

Notoriously, social cognition is one of the fields to which NI has been taken to be relevant. Since our critical examination of theories (see Explanations for the NI Findings) left us with two most promising perspectives on NI, we address the question of its implications for social cognition from those two perspectives. What is the significance of NI for social cognition if the arousal hypothesis is correct? Or if AST is?

In light of the arousal perspective, one can hypothesize a dynamic of this kind: adult facial movement causes infant arousal, arousal causes infant response, response affects parents, parents are encouraged to continue with facial games, etc. In this way, arousal could facilitate early social interaction. Therefore, arousal is naturally integrated in theories of the indispensability of affective interaction for the development of social cognition (Gallagher, 2001, 2005; Trevarthen and Aitken, 2001; Reddy, 2008) and empirical investigation on infant-caregiver interaction should seek to specify its role in more precise terms. On this matter, one question is whether parents are drawn into the interaction by the fact that they (over-) interpret infants’ responses as imitative. This is unlikely. Meltzoff and Moore (1977, p. 77) noted that “no parent was aware of ever having seen babies imitating […]; indeed, most were astonished at the idea.” And even another defender of the existence of differential imitation and its social-communicative function suggested that imitation affected parents without parent recognizing it as imitation (Heimann, 2002).^17^ Thus, if arousal contributes to social interaction, it is probably not because caregivers interpret infant responses as imitative, but because these responses have a different (perhaps more subtle) social meaning.

Heimann (2000, p. 181) raised the possibility that “NI exists, but plays no major role in early infant development”—although he did not endorse this view. As it should be clear from Sections “The Operational Definition and the Importance of Keeping it in Mind” to “Proposals for Experimental Design,” AST is consistent with such a hypothesis. Hence one may wonder whether, from the AST perspective, NI has any relevance to the field of social cognition. In this regard, we would like to emphasize two points. First, AST is compatible with arousal having a role in provoking infant responses, especially TP. Thus, anything that can be said about how arousal may contribute to social interaction can be happily accepted in AST. Moreover, AST does not exclude the idea that similarity-based induction may occur sometimes in real mother-infant interaction (outside an experimental context). If it occurs, the infant’s response will be characterized by timing and kinetic features apt to facilitate interaction, whether or not caregivers interpret the response as imitative.

Second, if AST were verified, it would support the Direct Social Perception (DSP) hypothesis (Gallagher, 2015). This hypothesis is part of a wider approach to social cognition called Interaction Theory (IT), which is proposed as an alternative to the traditional theories of mindreading, Theory-Theory (TT) and Simulation Theory (ST). In order to show how AST would support DSP, it is necessary to provide some background on DSP and Meltzoff’s version of TT.

Direct social perception relies on insights from the phenomenological tradition. In authors like Husserl and Merleau-Ponty, the dynamics operating in ordinary perception play a role in social perception as well. For example, Husserl (1931/1999) suggests that, after a child learns the meaning of scissors, she *directly* perceives a newly encountered pair as scissors. That is, the child does *not* have to (1) recall scissors experienced in the past, (2) compare the present object with objects experienced in the past, (3) recognize that the object is *like* some other scissors previously experienced, and (4) infer that the object is a pair of scissors. These mental acts (1–4) do not occur. Rather, perception of the current stimulus involves a reawakening of sensory-motor content (a reactivation of neuronal patterns) in virtue of the similarity between the stimulus and specific past experiences. More specifically, Husserl and other phenomenologists take this similarity to be pragmatic or affordance-based, defined in terms of what I can do with the object. The crucial point is that pragmatic similarity regulates how we perceive visual stimuli, but is not the object of a mental act—the act of recognizing the similarity between different objects (3) does not occur. The child simply sees a pair of scissors.

Now, just as we perceive the specific pragmatic meaning of an object on the basis of an association by similarity with past experienced objects, so can we perceive some mental states (such as action intentions, i.e., low-level motor intentions, or ‘intentions-in-action’) in others on the basis of the pragmatic similarity of their behavior with our own. This is not accomplished through a comparison of the visual image of the other with the experience of one’s own body. Rather, similar body-schematic (action production) processes involved in fulfilling our own action intentions are activated in perceiving the other’s action (Merleau-Ponty, 1964). I experience my bodily behaviors as intentional; thus, when I encounter the similar behaviors of others, I apprehend them in terms of associated intentions. Thus, DSP does not deny that social perception is affected by past experience (Gallagher, 2008; Zahavi, 2011, 2014). In opposition to TT and ST, however, DSP denies that social perception involves intermediary steps (e.g., the perception of “mere behavior” or a comparison between behaviors, theoretical inference, simulation routines) to infer mental states.

Consider how infants perceive the goal-directedness of others’ actions. A growing body of literature attests that action production influences action perception: mechanisms involved in action production allow infants to perceive others’ action as goal-directed, or intentional, behavior (Woodward, 1998; Sommerville and Woodward, 2005; Sommerville et al., 2005, 2008; Falck-Ytter et al., 2006; Hauf et al., 2007; Cannon et al., 2011; Hauf and Power, 2011; Filippi and Woodward, 2015). Meltzoff’s (2005, 2007, 2009; Gopnik and Meltzoff, 1997; Meltzoff and Decety, 2003) version of TT interprets this evidence for the role of action production in action perception by separating two cognitive operations. The first is the recognition that the other is “like me,” as evidenced in NI. The second is the inference that, because others behave “like me,” they have the kinds of mental states I have. In contrast, DSP can rely on the tacit functioning of pragmatic similarity. When the infant perceives others’ actions, it interprets them in light of its similar goal-directed behavior: similarity is not the object of a cognitive act, but the mechanism in virtue of which a specific content, i.e., goal-directedness, is awakened and put to use in perception. In DSP, others’ actions are not perceived as being “like my own actions.” Rather, they are *directly perceived as goal-directed.*

The difference between Meltzoff’s TT and DSP is not just verbal. The two theories require two different interpretations of imitation, postulate different psychological states and these postulations lead to different empirical predictions. Meltzoff’s TT is based on AIM. Recall that AIM assumes that imitation entails the recognition of self-other similarities, the thought “That seen event is like this felt event,” and this recognition is implemented by a specialized cognitive machinery evolved to provide infants with the “innate grasp that others are like me” (Meltzoff, 2002, p. 9). If AIM is verified, imitation entails the comparison and the recognition of similarity between actions of self and others. Hence Meltzoff’s TT is supported, because it becomes plausible to assume that later low-level mental state attribution is an inference grounded on recognition of similarities.

In contrast, if AST is verified, imitation does not entail comparison and similarity recognition. Thus DSP is strengthened in two ways: (a) imitation does not attest to a comparison/similarity recognition that can function as premise for inferences to mental states; (b) the idea that similarity operates in action perception without being the object of a psychological act is supported. That is to say, just as in NI similarity with one’s own behavior merely awakens specific action tendencies, so in action perception it simply awakens the pragmatic sense in reference to which actions are interpreted, i.e., goal-directedness.

In Section “Explanations for the NI Findings,” we argued that AIM is disqualified by the findings. Consequently, we submit that Meltzoff’s TT is undermined too. Yet AST is still a viable hypothesis and can contribute to validate DSP. AST shows that DSP is right in denying the need for postulating a specialized module for comparison/recognition of self-other similarities. Indeed, since AST relies on similarity as a domain-general process of association, DSP is supported in claiming that action production affects action perception in the same way as past experience influences ordinary perception. In perception, similarity awakens processes in light of which one comprehends the current stimulus. No intermediary act of comparison with past experiences is required.

For these reasons, from the AST perspective NI has important significance for theorizing on social cognition. It can lend support to theories of social interaction and to DSP.

## Conclusion

As it has been noted many times, questions about NI have implications for neuroscience, the development of imitation and social cognition, and for the inquiry into the general relationships between action and perception. In this paper, we have discussed the importance of keeping in mind the operational definition of NI as differential imitation for more than one gesture. This definition contains key reminders: differential imitation neutralizes the arousal explanation; a mere increase of one gesture with respect to baseline does not count as evidence for imitation; and NI does not look like prototypical imitation. We examined explanations for the findings and argued that two of them, the arousal hypothesis and AST, have the best chances for success. The latter hypothesis is a consistent application of the ideomotor approach (Prinz, 1990, 1997, 2005), and sees differential imitation as differential induction of spontaneous behavior through similarity. We then outlined experimental designs aimed at testing the AST hypothesis. In contrast with Oostenbroek et al.’s (2016) symptomatic study, AST leads experimenters to implement procedures that may facilitate differential induction and its detection: affective-attentional control, a sample size constrained for maximal internal validity, taking each infant as its own control, employing a limited number of control models and only one model in each session, comprehensive response phases, strictly regulated settings, repetition of the same stimulus across different days (in the One-Model design), analysis of the correlation between spontaneous production and imitation, and of the behavior of individual infants. Moreover, AST makes specific predictions for different models, shifting the focus to MO and HR. We concluded by considering the relationship between the topic of NI and the field of social cognition. We noted how, from the point of view of AST, the NI findings are naturally integrated in theories of social interaction and support the particular way in which the DSP hypothesis can account for the effect of action production on action perception.

## Author Contributions

SV is lead author and wrote the final version. YJ, EB, and SG reviewed and accepted the final version. All authors contributed to the theoretical formulations.

## Conflict of Interest Statement

The authors declare that the research was conducted in the absence of any commercial or financial relationships that could be construed as a potential conflict of interest.

## Footnotes

1 In this paper, we adopt this terminology. Primarily, we use the expression “neonatal imitation" (NI) as shorthand for “differential imitation in the first 2 months of post-natal life." At times, however, we use the expression more broadly to refer to the relevant empirical literature, whether or not this literature actually provides evidence for differential imitation.

2 Meltzoff and Moore (1994, p. 83): “In order to imitate, the child must see the adult’s actions, use this visual perception as a basis for an action plan, and execute the motor output” (see also Prinz et al., 2009, p. 48).

3 In this paper we follow the extant literature in using the term ‘representation.’ We take this term to be equivalent to a pattern of neural activation that co-varies with a relevant stimulus or experience.

4 See Meltzoff (1999, 2002, 2005, p. 254; p. 24; p. 72) and Meltzoff and Moore (1997, 1999 p. 180 and 182, p. 52).

5 Meltzoff and Moore (1997, p. 188; cf. p. 185) assume that the “representation of the other’s body is separate from [the] representation of the infant’s body.”

6 See Kugiumutzakis (1999) for an exception.

7 See studies in footnote 2.

8 Indeed, theories of perception in philosophy and cognitive science acknowledge that present stimuli are apprehended in light of past perceptual experiences that presented commonalities with the current stimuli (Barsalou, 2008; Meyer and Damasio, 2009; Clark, 2013; Vetter and Newen, 2014).

9 In addition to the textual evidence provided in Section “Active Intermodal Matching,” we should mention that this is how AIM is usually understood (Heyes and Ray, 2004; Heyes, 2016).

10 Prinz presented the basic assumptions of common coding and ideomotor theory in texts from 1990 to 2009 (cited above). However, in the context of broad speculations on the nature of mind, agency and sociality, Prinz (2012) offers a “reinterpretation” of common coding, which cannot be considered as just another presentation of the same theory (*pace* Prinz). We believe that this reinterpretation is unwarranted and jeopardizes the most meaningful insights of the common coding approach. The common coding approach is guided by phenomenological analysis and defined event and action codes in terms of their function of representing “environmental events,” i.e., events in the (spatiotemporal) world. The overlap (or communication) between perception and action is just the consequence of the fact that perception and action can share characteristic features (cf. Prinz, 1990, 1997, pp. 171–172, pp. 131–133; Prinz et al., 2009, p. 39). In contrast, Prinz (2012, pp. 66–68) abandons his previous phenomenological orientation and defines action and event codes though their role in a “hybrid view” that takes separate coding and arbitrary couplings to constitute the basic workings of the mind, and common coding as appearing in modules engineered to remedy the incommensurability of disparate coding. In particular, it is assumed that these modules evolved specifically for socio-cognitive functions (cf. Herwig et al., 2013, p. 15). The consequence of reinterpreting event and action codes as entries in a secondary system evolved on top of separate coding is the multiplication of “disparate languages” and “translation” processes. In addition to translation from the sensory to the motor, there is “increasing demand on disparate coding as it is required for translating information from the sensory and motor domains into the common coding domain” (Prinz, 2012, p. 68). In other words, the same kinds of translation processes that are required from sensory to motor codes are now required from sensory codes to event codes and from action codes to motor codes. In Prinz’s (2012) hybrid view, it is true not only that perception and action talk two disparate languages, but also that there are disparate languages even within perception and action, respectively! This is the opposite of the logic proposed in Prinz (1990, 1997), where common coding was presented as a more appropriate account precisely because it did not require arbitrary translation processes for relating perception and action.

11 Vincini and Jhang (unpublished) provide a list of studies attesting that the frequency of the actions in question before birth (in particular during the third trimester of pregnancy) is comparable to their frequency after birth. Cf. Keven and Akins, 2016, section 7: “8 weeks before birth the behavioral repertoire of the neonate is in place ready for post-natal life and all of the gestures tested in imitation experiments come from this repertoire.”

12 Their frequencies of spontaneous execution are approximately 1.85 and 1.15/per minute, respectively. The number for TP was retrieved and averaged across the following studies: Heimann and Schaller, 1985, Heimann et al., 1989, Meltzoff and Moore, 1989, Ullstadius, 1998, Nagy et al., 2013; that for MO across the following: Heimann and Schaller, 1985, Heimann et al., 1989, Coulon et al., 2013. Compare these frequencies with that of, for example, spontaneous lip movements, i.e., 0.14 per minute (Ekman and Rosenberg, 1997). Frequencies for the other gestures can be found in other studies and are significantly lower than those for TP and MO (Meltzoff and Moore, 1989; Kurjak et al., 2004; Oostenbroek et al., 2016).

13 See Heimann (2000, p. 185) for evidence for the correlation between attention and imitation.

14 Oostenbroek et al. (2016, Supplemental Materials) report: “If the infant became sleepy or upset, testing was paused and calming methods such as rocking, jiggling or walking the infant around the room were used to bring the infant back to a quiet alert state.” However, an infant who continues with the test after having cried or fallen asleep is not in the same affective state as one who starts the experiment fresh. Long testing sessions like those in Oostenbroek et al. (2016) make it difficult to keep the infant affective state constant and optimal for the entire test.

15 Anisfeld (2005; Anisfeld et al., 2001) raises the issue that the order of conditions in one experimental session may affect TP production. One may think that this is potentially a problem for the Many-Models design just proposed because TP increase with respect to baseline may be due to the order of these conditions (although we remind the reader that AST does not put much emphasis on TP and so, if imitation is attested for another gesture, e.g., MO, AST is supported). If one decides to give weight to Anisfeld et al.’s (2001) findings, there is a simple way to eliminate the possible effect of the order of conditions. It is sufficient to eliminate the PF condition before the test phases for the other models and add a further session on a separate day in which the PF condition is treated just like the other test phases. In this way, all conditions count as conditions occurring first. This is a valid alternative design because it has the advantage of making experimental sessions shorter.

16 Features of the modeler that may distract infants should be avoided. For example, glasses or excessive hair are salient to infant (Meltzoff and Moore, 1994) and may distract them from the models.

17 Heimann (2002) hints at the idea that, if some parents today think that neonates imitate them, it is because research on NI has been widely popularized.
